# Effect of Alkalinity on Catalytic Activity of Iron–Manganese Co-Oxide in Removing Ammonium and Manganese: Performance and Mechanism

**DOI:** 10.3390/ijerph17030784

**Published:** 2020-01-27

**Authors:** Ya Cheng, Shasha Zhang, Tinglin Huang, Feifan Hu, Minyi Gao, Xiruo Niu

**Affiliations:** 1 Key Laboratory of Northwest Resource, Environment and Ecology, MOE, Xi’an University of Architecture and Technology, Xi’an 710055, China; chengya.xauat@outlook.com (Y.C.); silenceee26@163.com (S.Z.); hufeifanhhh@163.com (F.H.); 18628613581@163.com (M.G.); niuxiruoajiu@163.com (X.N.); 2 Shaanxi Key Laboratory of Environmental Engineering, Xi’an University of Architecture and Technology, Xi’an 710055, China

**Keywords:** Fe–Mn co-oxide, filter media, surface water, alkalinity, activity recovery

## Abstract

In this study, a pilot-scale experimental filter system was used to investigate the effect of bicarbonate alkalinity on the activity of an Fe–Mn co-oxide for ammonium and manganese removal from surface water. The results showed that an increase in alkalinity to 150 mg/L (calculated as CaCO_3_) by the addition of NaHCO_3_ significantly promoted the activity of the Fe–Mn co-oxide. The ammonium and manganese removal efficiencies of the Fe–Mn co-oxide increased from 40% to 95% and 85% to 100%, respectively. After NaHCO_3_ was no longer added, the activity of the filter column remained. Moreover, pH (7.4–8.0) and temperature (12.0–16.0 °C) were not the main factors affecting the activity of the filter, and had no significant effect on the activity of the filter. Further characterization analysis of the Fe–Mn co-oxide filter film showed that after alkalinity was increased, the accumulation of aluminum on the filter media surface decreased from 3.55% to 0.16% and the oxide functional groups changed. This was due to the action of bicarbonate and the residual aluminum salt coagulant in the filter, which caused the loss of Al from the surface of the filter media and weakened the influence of the aluminum salt coagulant on the activity of the Fe–Mn co-oxide; hence, the activity was recovered.

## 1. Introduction

In recent years, ammonium (NH_4_^+^) and manganese (Mn^2+^) have been common pollutants in surface waters, which have deleterious impacts on drinking water treatment plants as well as posing a severe threat to people’s health [1,2,3,4]. Excessive NH_4_^+^ in drinking water is undesirable and may cause numerous problems: (1) NH_4_^+^ contamination can create issues of taste and odor in water as well as eutrophication of surface waters [5,6,7]; (2) NH_4_^+^ not only decreases the efficiency of chlorine disinfection but also leads to the generation of toxic disinfection byproducts [5]; (3) excessive intake of NH_4_^+^ may be oxidized to nitrates (NO_3_^−^) and nitrites (NO_2_^−^), which can produce carcinogenic nitrosamines [8,9,10]. Manganese is present as Mn^2+^, the oxidation of which results in the formation of a dark brown MnO_2_ precipitate that can result in the discoloration of water or cause pipe blockages [11,12]. Mn in drinking water has also been linked to neurotoxic effects in children [13,14] and might affect the health of consumers [15,16,17]. At present, the general methods to remove NH_4_^+^ during drinking water treatment are adsorption [18], chemical oxidation [19,20,21], biofiltration processes [22], and membrane separation technology [23]. Meanwhile, many treatment options have been explored for Mn^2+^ removal, which include chemical oxidation [1] and biological processes [24,25,26]. However, the existing technologies have faced continued problems, including the high cost of oxidants and the poor applicability of microorganisms; hence, it is necessary to develop an affordable process for the efficient removal of NH_4_^+^ and Mn^2+^ in surface water.

Our research group recently reported on an iron–manganese (Fe–Mn) co-oxide filter media for NH_4_^+^ and Mn^2+^ removal from groundwater [27,28]. Given the lack of groundwater resources in China and the fact that most drinking water treatment plants still use surface water, it was used for surface water treatment. However, the aluminum (Al) deriving from the polymeric aluminum ferric chloride coagulant was found to negatively affect the NH_4_^+^ catalytic activity [29]. Specifically, the activity of the Fe–Mn co-oxide filter media decreased gradually when used for surface water treatment, which was termed “inactivated filter media”. Hence, the aim of this study is to recover the removal activity of the inactivated Fe–Mn co-oxide filter media used for surface water treatment. By comparison, the bicarbonate (HCO_3_^−^) alkalinity was found to be the parameter that differed the most between the groundwater and surface water. In the present study, we systematically investigate the NH_4_^+^ and Mn^2+^ removal efficiencies and the effects of HCO_3_^−^ alkalinity on the chemical catalytic oxidation of the Fe–Mn co-oxide filter. Water quality parameters and filter media characterization are analyzed to investigate how the catalytic activity is restored by alkalinity.

## 2. Materials and Methods

### 2.1. Experimental Setup

**Figure 1:**
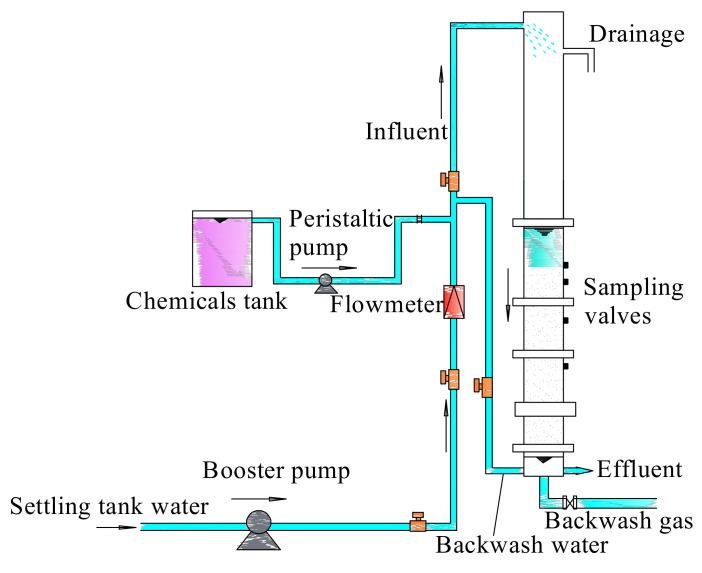
Schematic diagram of the pilot-scale experiment system.

A pilot-scale filter system was built at a drinking water plant located in Xi’an City, China. The pilot system consisted of a transparent plexiglass column with an inner diameter of 10 cm and a height of 270 cm, within which the Fe–Mn co-oxide filter media were packed to a height of 90 cm. The bottom of the filter column was supported by a 15 cm high layer of cobblestone, and a water level of 30–40 cm was maintained in the upper part of the filter layer during operation. Five sampling points were distributed along the depth of the filter column, located at 0, 10, 30, 50, and 90 cm (from top to bottom), as illustrated in Figure 1. The characteristics of the Fe–Mn co-oxide filter media that filled the filter and the backwashing parameters of the filter were consistent with our previous research [30,31,32].

Raw, untreated surface water was the influent of the surface water treatment plant. The feed water of the filter system was the effluent from a sedimentation tank in the pilot-scale treatment system. The parameters of the feed water are listed in Table 1. The filter was operated at a filtration rate of 6–7 m/h in downflow.

### 2.2. Experimental Methods

Given the relatively low NH_4_^+^ and Mn^2+^ concentrations in the surface water, the metering pump was adapted to dose a certain concentration of NH_4_Cl and MnCl_2_ solution into the influent of the filter column to adjust the Mn^2+^ and NH_4_^+^ concentrations (see below). In this study, the operation of the pilot-scale filter column was divided into four phases: before adding NaHCO_3_ (phase I: days 0–8), maintaining 100 mg/L alkalinity (phase II: days 9–19), maintaining 150 mg/L alkalinity (phase III: days 20–38), and alkalinity no longer added (phase IV: days 39–50). Sodium bicarbonate (NaHCO_3_) was used to adjust the alkalinity (calculated as CaCO_3_) in the filter. At each phase, the influent NH_4_^+^ and Mn^2+^ concentrations were maintained at 1–2 mg/L, and the NH_4_^+^ and Mn^2+^ removal efficiencies of the filter media at different phases were measured. Specifically, the ammonium and manganese removal efficiency of the filter was calculated according to Equation (1) [33]:

(1) $$\mathrm{removal}\;\mathrm{efficiency}(\%)=\frac{c_{\mathrm{influent}}-c_{\mathrm{effluent}}}{c_{\mathrm{influent}}}\times 100\%$$

where c is the concentration (mg/L).

### 2.3. Analytical Methods

During the pilot-scale system experiment, the effluent samples taken from different heights of the filter column were filtered through a 0.45 μm membrane filter. The pH, temperature, alkalinity, and NH_4_^+^ and Mn^2+^ concentrations of the effluent were measured daily. The pH was determined using a PHS-3C pH meter. NH_4_^+^, Mn^2+^, and alkalinity were measured according to the guidelines set by the Ministry of Environmental Protection of China [34]. Specifically, the concentrations of NH_4_^+^ and Mn^2+^ were measured by Nessler’s reagent spectrophotometry and potassium periodate oxidative spectrophotometry, respectively.

To explore the variations in morphology, structure, and composition of the Fe–Mn co-oxide filter film during the different operation phases, the following methods were used to periodically analyze the characteristics of the oxide film. The microtopography of the Fe–Mn co-oxide was measured using scanning electron microscopy (SEM) equipment (FEI Quanta 600F). The elemental composition of the samples was determined by energy dispersive X-ray spectroscopy (EDS) equipment (Oxford INCA/ENERGY-350). The specific surface area and pore size of the filter material was measured by Brunauer–Emmett–Telle (BET) equipment (Quantachrome Autosorb iQ2). The microstructure of the samples was analyzed using Fourier transform spectroscopy (FTIR) (chemical bond) equipment (Nicolet iS50) and X-ray diffraction (XRD) (crystal structure and crystallinity) equipment (Ultiman IV).

## 3. Results and Discussion

### 3.1. Effect of Alkalinity on NH_4_^+^ Removal Performance of the Filter System

**Figure 2:**
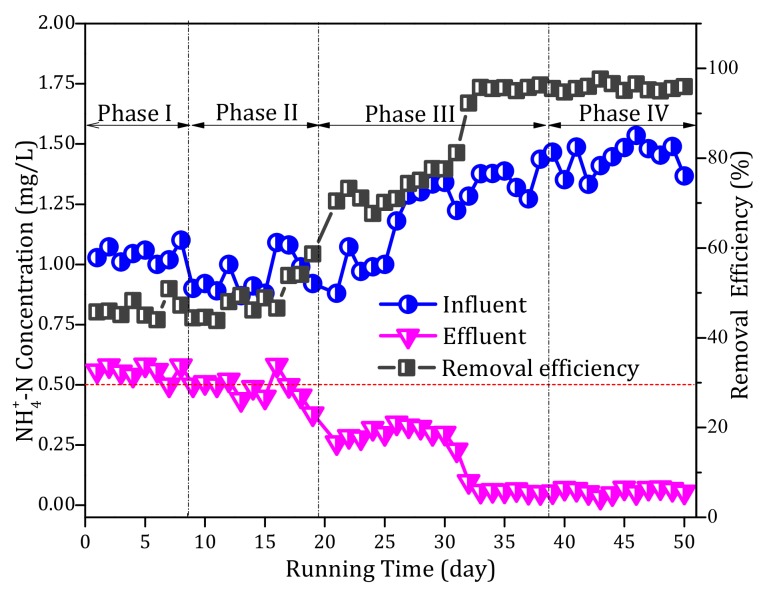
Profiles of the ammonium (NH_4_^+^) removal performance over the continuous operational period of the pilot filter system.

The pilot-scale filter system was operated continuously for 50 days. The NH_4_^+^ concentration in the influent and effluent at different phases is shown in Figure 2. During phase I, the influent alkalinity was the original alkalinity of the surface water (50–65 mg/L), and the NH_4_^+^ removal performance of the filter was always poor. In particular, when the influent NH_4_^+^ concentration was 1 mg/L, the effluent NH_4_^+^ concentration was ~0.6 mg/L and the removal efficiency was only 40%. After the influent alkalinity was increased to 100 mg/L (phase II), although the NH_4_^+^ removal efficiency increased, it did not change significantly. During phase III, the influent alkalinity was 150 mg/L and the influent NH_4_^+^ concentration was increased from 1 to 1.5 mg/L; the results showed that the effluent NH_4_^+^ concentration decreased gradually over time during phase III (Figure 2). From the 30th day, the effluent NH_4_^+^ concentration was < 0.1 mg/L, and the NH_4_^+^ removal efficiency subsequently improved to be consistently > 95%. The results showed that the increase in alkalinity was beneficial to the removal of NH_4_^+^ by the filter. To further explore whether the NH_4_^+^ removal activity of the filter was maintained, alkalinity was not added during phase IV. The filter system continued to exhibit an excellent NH_4_^+^ removal performance; even if the influent NH_4_^+^ concentration was as high as 1.5 mg/L, no NH_4_^+^ was detected in the effluent and the removal efficiency was always > 95% (Figure 2). The experimental results demonstrated that the NH_4_^+^ removal ability of the filter was indeed improved compared with that before increasing alkalinity.

**Figure 3:**
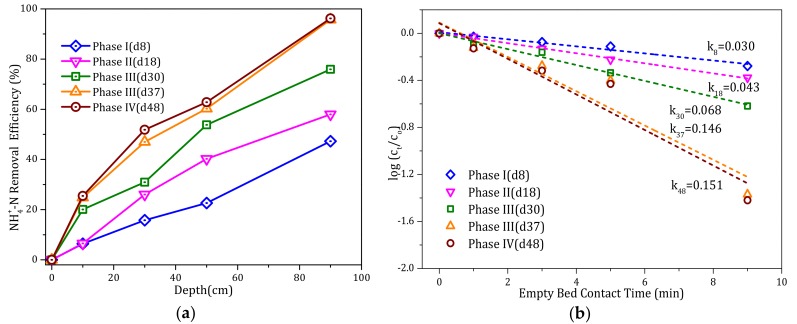
(**a**) Ammonium (NH_4_^+^) removal efficiency with filter column depth during different phases, and (**b**) linear regression analysis of NH_4_^+^ depletion with the empty bed contact time during different phases. Day (d) and rate constant (k).

Figure 3 presents the NH_4_^+^ removal efficiency with filter column depth during different operational phases and the corresponding kinetics of NH_4_^+^ oxidation. Figure 3a shows that as the influent alkalinity increased with each phase, the NH_4_^+^ removal efficiency at given depths improved significantly. At 30 cm depth, the removal efficiency was only 15% during phase I, but increased to 25% during phase II and then 47% during phase III, which was maintained during phase IV.

NH_4_^+^ was oxidized to nitrite and nitrate during its removal by the filter column (Equation (2)) [28]. The NH_4_^+^ removal kinetic was assumed to follow the pseudofirst order kinetic model expressed by Equation (3) [27]:

NH_4_^+^ + 2O_2_ → 2H^+^ + H_2_O + NO_3_^−^ (2)

(3) $$-\frac{d\left[{\mathrm{NH}}_{4}{}^{+}\right]}{dt}=k\left[{\mathrm{NH}}_{4}{}^{+}\right]$$

where k is the rate constant (min^−1^).

As shown in Figure 3b, the relationship between log{[NH_4_^+^]_t_/[NH_4_^+^]_o_} and the empty bed contact time (min) was linear. During phase I, the value of k_8_ (the rate constant on day 8) in the filter column was 0.030 min^−1^. As alkalinity was increased, the k value increased significantly to 0.146 min^−1^ on day 37 (phase III) and then further to 0.151 min^−1^ on day 48 after ceasing to add alkalinity (phase IV). This implies that the addition of alkalinity was beneficial for continuous NH_4_^+^ removal by the filter column.

### 3.2. Effect of Alkalinity on Mn^2+^ Removal Performance of the Filter System

**Figure 4:**
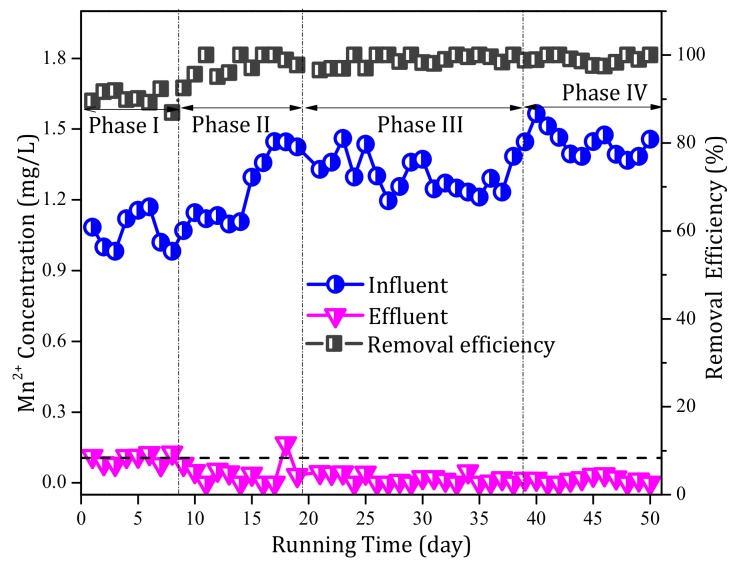
Profiles of the manganese (Mn^2+^) removal performance over the continuous operational period of the pilot filter system.

During the continuous operation of the pilot-scale filter system, the Mn^2+^ concentration in the influent and effluent were measured, as shown in Figure 4. During phase I, the influent alkalinity was 50–65 mg/L and the Mn^2+^ removal performance was always good. Specifically, when the influent Mn^2+^ concentration was 1 mg/L, the effluent Mn^2+^ concentration remained more or less constant and was always close to the limit of China’s drinking water quality standards (0.1 mg/L). The removal efficiency during phase I was ~85% (Figure 4), which improved considerably during phase II (after 10 days of operation) and reached up to 100% after the influent alkalinity was increased to 100 mg/L. When the influent Mn^2+^ concentration was increased from 1 to 1.5 mg/L during phase II, despite a small increase on day 18, the Mn^2+^ concentration in the effluent subsequently reduced to near zero. During phase III, when the influent alkalinity was increased to 150 mg/L, the Mn^2+^ concentration in the effluent remained near zero and the removal efficiency was consistently 100%. In addition, during phase IV, when no further alkalinity was added, the filter still showed an excellent Mn^2+^ removal performance, and the Mn^2+^ concentration in the effluent remained near zero (Figure 4). Hence, although the effect of alkalinity on the Mn^2+^ removal rate was not obvious, the Mn^2+^ removal capacity of the filter media increased from 1 to ≥ 1.5 mg/L.

**Figure 5:**
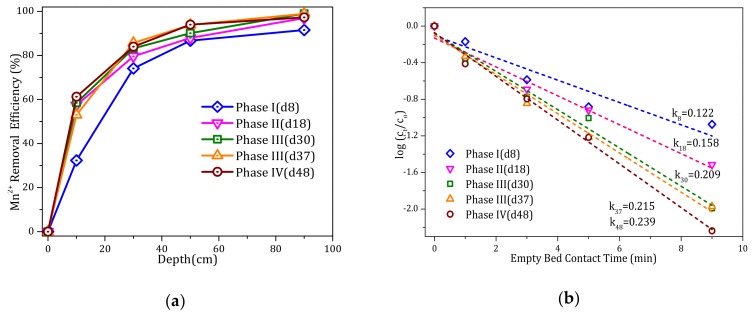
(**a**) Manganese (Mn^2+^) removal efficiency with filter column depth during different phases, and (**b**) linear regression analysis of Mn^2+^ depletion with the empty bed contact time during different phases. Day (d) and rate constant (k).

Figure 5 illustrates the Mn^2+^ removal efficiency over the depth of the filter column during different phases and the kinetics of Mn^2+^ oxidation. From phase I to phase II, as the influent alkalinity was increased, the Mn^2+^ removal efficiency increased considerably at given depths. In particular, the removal efficiency increased from 30% to 55% at the depth of 10 cm. After phase II, the Mn^2+^ removal efficiency tended to be stable, and the filter media maintained an excellent Mn^2+^ removal performance when alkalinity was no longer added in phase IV.

During the removal of Mn^2+^ from the filter column, the dissolved Mn^2+^ was converted into an insoluble manganese oxide and removed according to Equation (4) [32]. When the dissolved oxygen and pH remained invariable, Mn^2+^ depletion followed the first-order linear rate (Equation (5)) [35,36]:

2Mn(Ⅱ) + O_2_ + 2H_2_O → 2Mn(Ⅳ)O_2_ + 4H^+^ (4)

(5) $$-\frac{d\left[{\mathrm{Mn}}^{2+}\right]}{dt}=k\left[{\mathrm{Mn}}^{2+}\right]$$

where k is the rate constant (min^−1^).

As shown in Figure 5b, the relationship between log{[Mn^2+^]_t_/[Mn^2+^]_o_} and the empty bed contact time (min) was linear. During phase I, the value of k_8_ in the filter was 0.122 min^−1^. With increased alkalinity, the k value increased gradually to 0.215 min^−1^ on day 37 (phase III) and increased further to 0.239 min^−1^ on day 48 after ceasing to add alkalinity (phase IV). This indicated that the Mn^2+^ removal performance of the filter column was maintained. The results therefore demonstrated that the increase in alkalinity had a positive effect on the Mn^2+^ removal activity.

### 3.3. Variation of Other Water Quality Parameters

The experimental results presented in Section 3.1 and Section 3.2 showed that the increase in alkalinity had a favorable influence on the NH_4_^+^ and Mn^2+^ removal capabilities of the Fe–Mn co-oxide. A previous study found that the activity of Fe–Mn co-oxide was inhibited to a certain extent at low temperatures [37]. Meanwhile, the removal activity of Fe–Mn co-oxide was shown in another study to be affected by pH [38]. To further investigate the reasons for the difference in the activity of the filter during different stages, the influent temperature and pH of the filter were compared.

#### 3.3.1. Temperature

**Figure 6:**
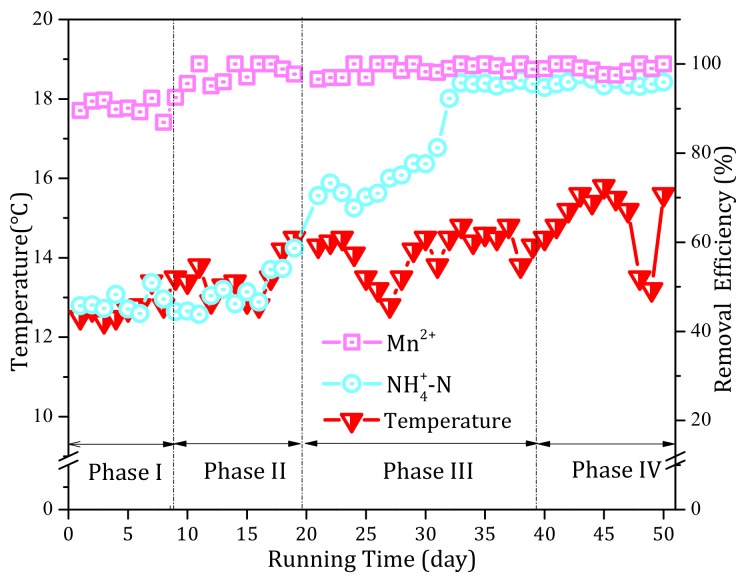
Profiles of the influent temperature during the continuous operational period of the pilot filter system.

Figure 6 exhibits the variations in the influent temperature of the filter over the entire operational period. The influent temperature tended to rise over the period of operation. During phase II, the gradual improvement in the NH_4_^+^ and Mn^2+^ removal performances should have been independent of temperature as a result of the fluctuating temperature (12–14 °C) during this stage. Similarly, between days 23 and 27 of phase III, the temperature decreased obviously and the NH_4_^+^ removal performance continued to improve. During phase IV, when the temperature dropped significantly, the NH_4_^+^ and Mn^2+^ removal capacities remained stable. The results therefore demonstrated that temperature was not the main reason for the enhancement of the removal capability of the filter system over the entire operation period.

##### 3.3.2. pH

**Figure 7:**
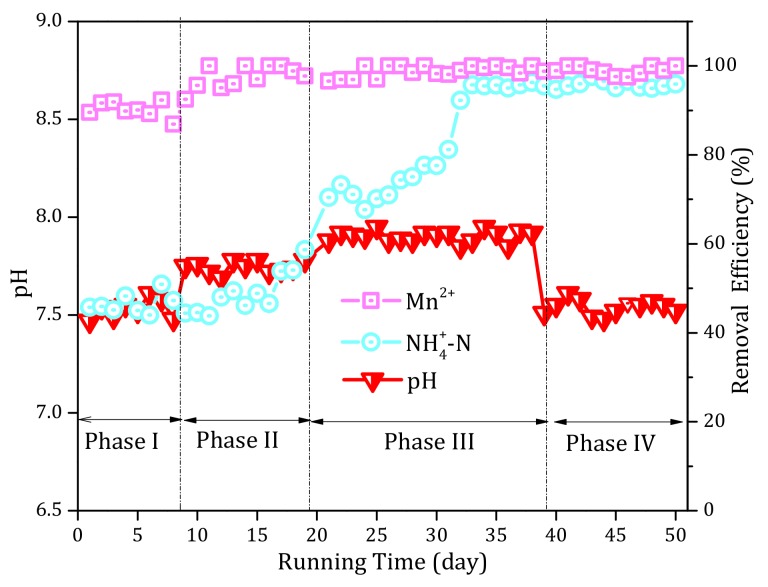
Profiles of the influent pH during the continuous operational period of the pilot filter system.

Figure 7 presents the influent pH values of the filter over the entire operation period. During phase I, the influent pH of the filter was ~7.5. When the alkalinity was increased to 100 mg/L (phase II) and 150 mg/L (phase III), the pH gradually increased to 7.7 and 7.85, respectively. Hence, with increasing alkalinity, the influent pH also increased. This was consistent with the improved NH_4_^+^ and Mn^2+^ removal efficiencies; hence, it is possible that the increased NH_4_^+^ and Mn^2+^ removal efficiencies might have arisen from pH. However, the pH decreased to 7.5 after ceasing to add alkalinity (phase IV), and yet the NH_4_^+^ and Mn^2+^ removal performance remained superior. Therefore, pH was not the main cause for the improved NH_4_^+^ and Mn^2+^ removal performances of the filter system.

### 3.4. Characterization of Fe–Mn Co-oxide Film

#### 3.4.1. Morphology Analysis

**Figure 8:**
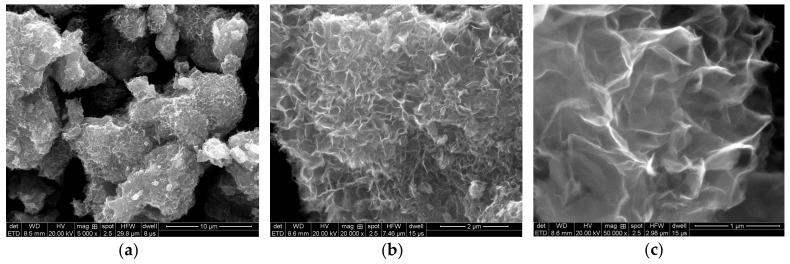

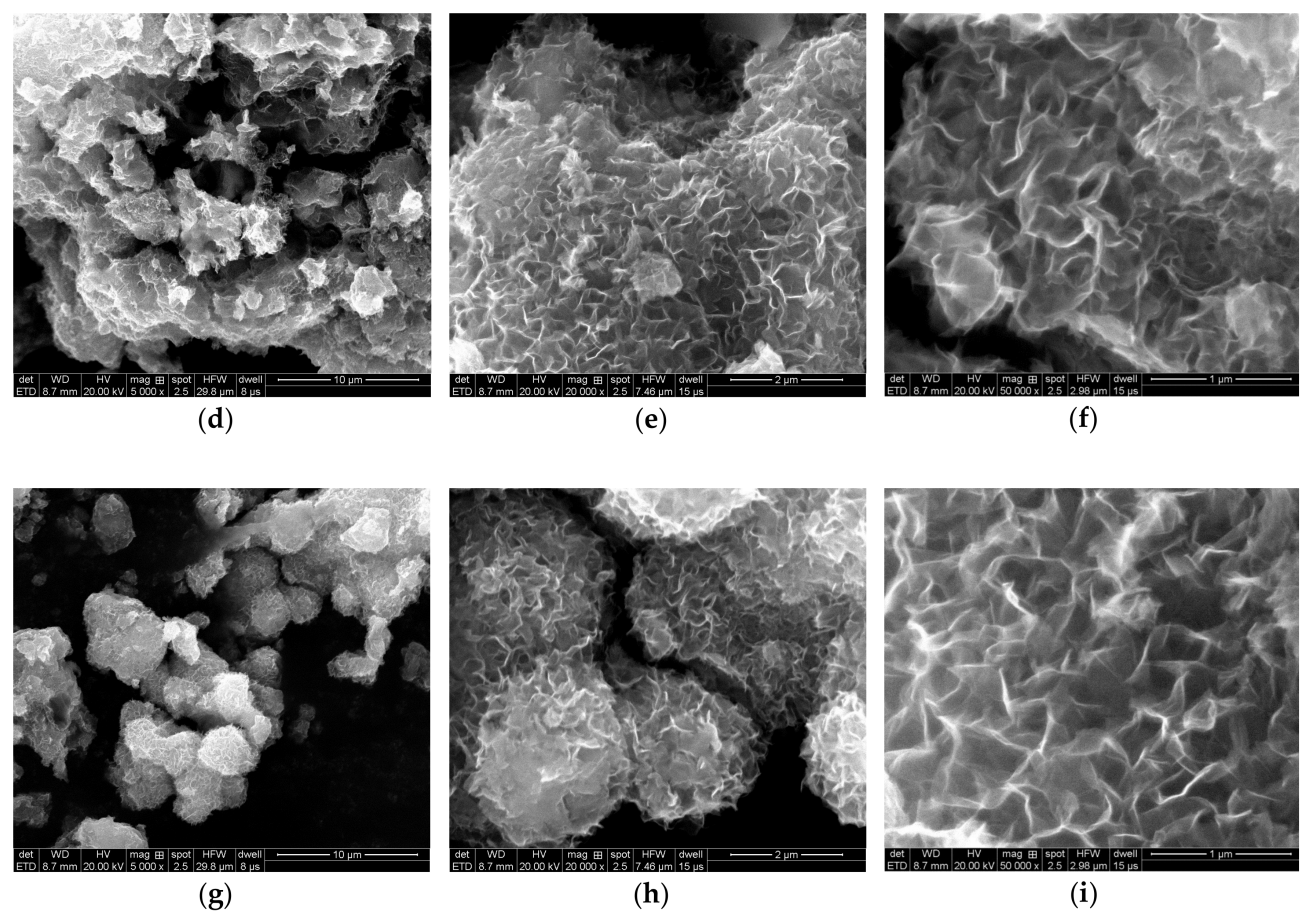
Scanning electron microscope (SEM) images of Fe–Mn co-oxide filter media during (**a**, **b**, and **c**) phase I; (**d**, **e**, and **f**) phase III; (**g**, **h**, and **i**) phase IV.

The build-up and surface microtopography of the Fe–Mn co-oxide at different phases during the entire operational period were analyzed by SEM, as shown in Figure 8. The images obtained from the Fe–Mn co-oxide sample showed that the media consisted of many small aggregated particles (Figure 8a,d,g), which led to a rough surface and a porous structure. Adsorption onto the oxide film should be the first step for the removal of NH_4_^+^ and Mn^2+^ by the Fe–Mn co-oxide, such that the relatively loose structure could facilitate the transfer and degradation of NH_4_^+^ and Mn^2+^. Moreover, it can be seen that the surface of the filter media exhibited relatively rough folds (Figure 8b,e,h), which were formed by the Fe–Mn co-oxide during the removal of pollutants adhered to the surface of the filter media. During phase I, the surface was reticular (Figure 8b,c) and could facilitate the contact and adsorption of pollutants; furthermore, NH_4_^+^ and Mn^2+^ were removed by the catalytic oxidation of the filter media. However, on the basis of the reticular structure, the surface of the filter media during phase III showed more folds (Figure 8e,f) that provided more adsorption sites, which was consistent with the increased removal activity with increased alkalinity during this stage. In addition, there was no significant difference in morphology between phases III and IV (Figure 8f,i), which was consistent with the well-maintained activity of the filter after ceasing to add alkalinity.

In addition, BET was adopted to analyze the surface area (S_BET_), pore volume (V), and pore size (d) of the samples, and the results are listed in Table 2. The surface areas of the samples collected from phase I, phase III, and phase IV were 8.04, 16.46, and 18.26 m^2^/g, respectively. The larger contact surface area implied that there are more adsorption sites on the surface, and the increase in pore volume indicated an increase in reaction channels used to remove contaminants. The pore volume of the filter media collected from phase III and phase IV was larger than that of phase I, thus indicating that an increase in alkalinity could increase the reaction channel for removing contaminants. After increasing the alkalinity, the gradually increasing mesoporous structure was conducive to adsorbing NH_4_^+^ and Mn^2+^ to the surface of the Fe–Mn co-oxide filter film for effective removal. Furthermore, the catalytic activity remained excellent even after ceasing to add HCO_3_^−^.

#### 3.4.2. XRD Analysis

**Figure 9:**
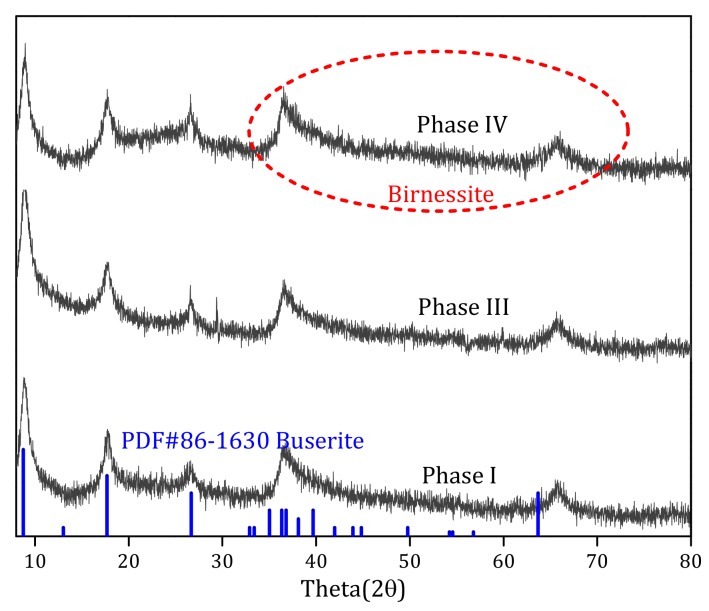
X-ray diffraction (XRD) analysis of the Fe–Mn co-oxide filter media at different operational periods.

To identify the crystal structure and crystallinity of the Fe–Mn co-oxide, samples from different phases were characterized by XRD. As demonstrated by Figure 9, the XRD patterns generally showed low peak intensities and broad diffraction peaks, which indicated a low crystallinity and a higher number of adsorption sites in the Fe–Mn co-oxide samples. These results agreed with the previous studies, which had a mixed phase structure (birnessite, buserite PDF#86-1630) [28,39]. Specifically, at different stages of operation, the crystal form and phase of the filter media did not show any obvious change, which indicated that NaHCO_3_ had no influence on the structure of the Fe–Mn co-oxide film. It was also found that the crystal structure stability of the Fe–Mn co-oxide was always better.

#### 3.4.3. Composition Analysis

The major elemental composition of the Fe–Mn co-oxide filter media samples collected from the filter was characterized by EDS, the results of which are listed in Table 3. The analysis showed that large amounts of Mn and O combined with Fe, Ca, Si, and Al on the surface of the filter media. It is worth noting that Al is not an original constituent element of the active oxide film. Based on our previous research, after the active oxide film was applied to the surface water treatment, the decreased catalytic oxidation activity may have been caused by the Al accumulation on the surface of the filter media [30]. Table 3 shows that the Al accumulation on the filter media surface decreased from 3.55% before the addition of alkalinity to 0.16% after NaHCO_3_ was added. Therefore, it can be inferred that the action of the HCO_3_^−^ and the residual Al of the filter media resulted in the loss of Al from the surface of the filter media and weakened the influence of Al on the catalytic activity. These results were consistent with those of the NH_4_^+^ and Mn^2+^ removal performance (Section 3.1 and Section 3.2); the reduced residual Al resulted in a higher removal efficiency of the filter column.

#### 3.4.4. FTIR Analysis

**Figure 10:**
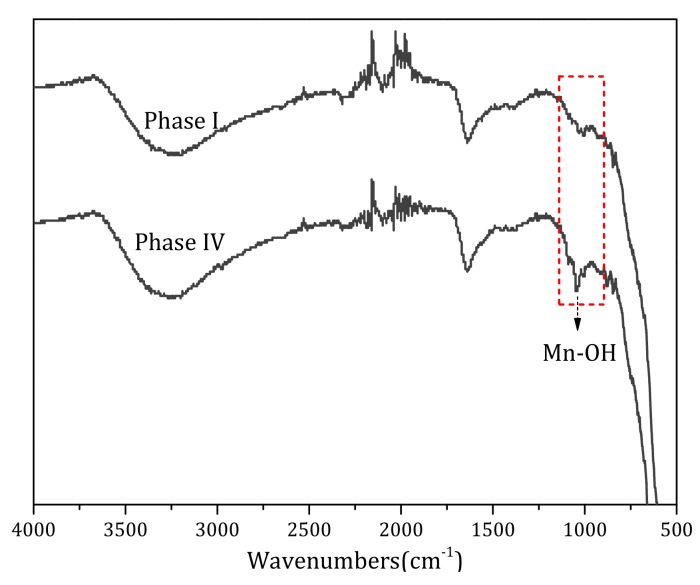
Fourier transform spectroscopy (FTIR) spectra of the Fe–Mn co-oxide during phases I and IV.

FTIR was used to explore the structural difference of the Fe–Mn co-oxide before and after adding NaHCO_3_, and the results are shown in Figure 10. There was no great difference among the oxide samples, which mainly contained three peaks: 3300, 1628, and 1037 cm^-1^. The broad peak at 3300 cm^−1^ was assigned to the stretching vibration of water molecules or –OH groups [40,41], whereas the peak at 1628 cm^−1^ was related to the bending vibration of structural water molecules or –OH groups [41,42], and the peak at 1037 cm^−1^ was linked to the bending vibration of the –OH group (υMn–O–H) [43]. However, it is noteworthy that after the addition of alkalinity, the surface structure of the oxide filter media changed and the intensity of the peak at 1037 cm^-1^ was obviously enhanced. This indicates that the Mn–OH bond in the Fe–Mn co-oxide was restored in a high alkalinity environment. Combined with the results of the EDS, the accumulation of Al decreased significantly with the increased catalytic activity of the filter media. Therefore, it was speculated that the HCO_3_^−^ alkalinity promoted the recovery of Mn–OH bonds, and the recovery of catalytic activity was attributed to the combination of HCO_3_^−^ and Al.

### 3.5. Mechanism of the Influence of Alkalinity on the Activity

**Figure 11:**
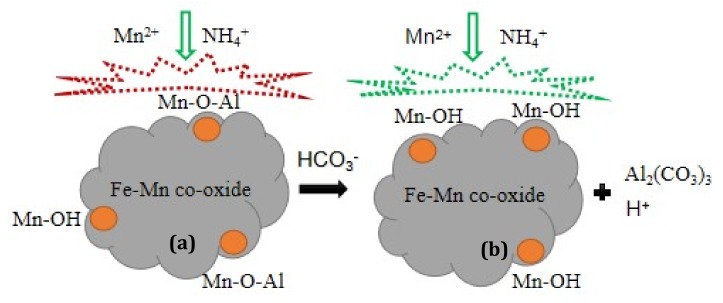
Mechanism of the influence of alkalinity on the NH_4_^+^ and Mn^2+^ removal activity of Fe–Mn co-oxide, (**a**) inactivated, and (**b**) active Fe–Mn co-oxide filter media.

The results in Section 3.1, Section 3.2, Section 3.3 and Section 3.4 collectively suggest that the NH_4_^+^ and Mn^2+^ removal activities of the inactivated filter media were recovered after operation in high alkalinity conditions, and that temperature and pH were not the reasons for the recovery of activity. The oxidation process of NH_4_^+^ produced H^+^, which reduced the pH and inhibited the further progress of the oxidation of NH_4_^+^. Therefore, the increased alkalinity buffered a decrease in pH and promoted the oxidation of NH_4_^+^, such that the activity of the filter column was promoted during the addition of alkalinity. The FTIR and EDX analysis illustrated that after a period of time in high alkalinity conditions, the functional groups and Al content of the filter media changed significantly. This was mainly due to the action of HCO_3_^−^ and the remaining Al salt coagulant in the filter, which caused the residual Al on the surface to fall off, thus leaving the active sites on the surface of the filter media exposed and meaning the removal activity was recovered finally. Specifically, the removal of NH_4_^+^ and Mn^2+^ is closely related to the –OH groups on the surface of the Fe–Mn co-oxides [26,40]. The possible mechanisms by which the alkalinity affected the activity of the Fe–Mn co-oxides is summarized in Figure 11. The active sites (–OH groups) on the surface of the inactivated Fe–Mn co-oxides (Figure 11a) were occupied by the remaining Al. After increasing alkalinity, Mn–O–Al combined with HCO_3_^−^ to form Al_2_(CO_3_)_3_, which caused Al to fall off and the active sites to be exposed (Figure 11b). Thus, the NH_4_^+^ and Mn^2+^ removal activities were recovered and remained superior even after ceasing to add HCO_3_^−^.

## 4. Conclusions

In this paper, the effects of HCO_3_^−^ alkalinity on the catalytic properties of Fe–Mn co-oxide for NH_4_^+^ and Mn^2+^ removal from surface water were studied. The results suggest that the increase in alkalinity had a positive effect on the NH_4_^+^ and Mn^2+^ removal activities of the inactivated filter media. In addition, after a period of operation under high alkaline conditions, the high activity of the filter column remained even if NaHCO_3_ was no longer added. Analysis of the filter media characterization indicated that the increase in NH_4_^+^ and Mn^2+^ removal activities with the increase of alkalinity might have arisen from the structural change, which was caused by the actions of Al and HCO_3_^−^. These resulted in the re-exposure of the active sites occupied by residual Al and the recovery of the removal activity.

## Figures and Tables

**Table 1:** Water quality parameters of the feed water.

Parameter	Unit	Range
pH		7.0–8.0
Temperature	℃	10–20
Mg^2+^	mg/L	3–4
Ca^2+^	mg/L	25–28
SO_4_^2−^	mg/L	16–25
Cl^−^	mg/L	10–20
Turbidity	NTU	0.1–2.0
Dissolved oxygen	mg/L	6–7
NH_4_^+^-N^1^	mg/L	1–2
Mn^2^^+^	mg/L	1–2

^1^ NH_4_^+^-N: the concentration of NH_4_^+^ calculated as nitrogen element.

**Table 2:** Brunauer–Emmett–Telle (BET) analysis of the filter media samples collected from different phases.

	BET Surface Area (m^2^/g)	Pore Volume (cm^3^/g)	Pore Size (nm)
phase I	8.04	0.03	9.89
phase III	16.46	0.06	14.65
phase IV	18.26	0.07	14.75

**Table 3:** Surface elemental composition (%) of the Fe–Mn co-oxide during phases I and IV.

	O	Al	Si	K	Ca	Mn	Fe
Phase I	59.85	3.55	2.74	0.35	2.65	30.19	0.68
Phase IV	60.95	0.16	2.33	0.11	3.05	32.13	1.25
